# Ultraviolet exposure of mice fed a high fat diet reduces weight gain and markers of liver disease progression

**DOI:** 10.1038/s41366-025-01779-5

**Published:** 2025-04-28

**Authors:** Gareth Hazell, Marina Khazova, Hannah Mancey, Raymond Shek, Paul O’Mahoney

**Affiliations:** UK Health Security Agency, Chilton, Didcot, OX11 0RQ UK

**Keywords:** Metabolic syndrome, Translational research

## Abstract

**Background:**

Research suggests that ultraviolet (UV) exposure of mice placed on a high fat diet can reduce incidence of metabolic disease. However, current research had primarily focused on male mice with UV outside level of terrestrial sunlight.

**Objectives:**

Here we attempt to address this imbalance, with a pilot study presented wherein female mice C57Bl6 mice are included, with UV exposure at level comparable to low dose (non-burning) sunlight exposure.

**Methods:**

2% UV-B and 98% UV-At a dose of 1.83 J/cm^2^ with UV-A and 0.04 J/ cm^2^ UV-B were delivered over a 10-min timeframe twice weekly. Mice were placed on a low-fat diet or high fat diet, with the high fat diet cohort either exposed twice weekly to UV light or sham exposed.

**Results:**

Non-significant trends are observed for weight amelioration in UV exposed mice across both sexes at study endpoint, whereas in the liver, a reduction of lipid droplet size due to UV exposure is observed. Assessment of vitamin D status at study endpoint shows that the high fat diet increases 25(OH)D level in both sexes, more so in female mice, with further non-significant rises due to UV exposure.

**Conclusions:**

This study supports previous evidence that non-vitamin D mediated pathways may be responsible for the outcomes reported in this study. The UV exposures used in this study also resulted in minimal damage to ex vivo skin or in vitro cells, as assessed by cyclobutene-pyrimidine dimers (CPD’s) (characteristic signature mutations induced by UV), and double stranded breaks, further demonstrating the potential benefit of such exposures. This study supports and builds on current evidence that non-vitamin D pathways mediated through UV exposure may be beneficial in slowing weight gain and liver disease progression.

## Introduction

Ultraviolet light (UV) can regulate the function of organs far below the skin [1, 2]. This has been appreciated for over a century, with cardiovascular disease (through blood pressure reduction) and over a century via rickets (via vitamin D) most clearly evidenced [2–4].

More recently, evidence suggests that UV also has the potential to slow metabolic disease in rodent models, via stalling lipid uptake in adipose tissue and liver. This appears to be independent of vitamin D as the use of a vitamin D proficient diet (2000 IU / kg) does not slow weight gain [5, 6]. These studies instead cite norepinephrine and nitric oxide as potential mediators of this response. Downstream effectors ‘touched’ by these intermediates remain elusive, with evidence that increased energy expenditure, and not behavioural alteration through mechanisms such as reduced food intake, mediate this effect [5–9]. Quan et al. demonstrated this clearly suggesting that thrice weekly UV-B exposure (100–400 mJ/cm^2^) over a twelve-week timeframe increases food intake but mice weigh less through increased energy expenditure. Reduction in the adipokine leptin induces response to food intake [6, 9]. Conversely, thermogenic ‘browning’ of adipose tissue to a mitochondrial-rich form permits mitochondrial uncoupling protein-1 to break down fat more effectively following UV exposure [5, 6].

Reduction of liver steatosis by UV exposure is more complex, with vitamin D mediating this effect in oral dosing studies [10], and UV-based studies implying other pathways such as nitric oxide act as mediators in vitamin D insufficient mice [2, 5]. This effect is shown in male mice only (understood to be non-responsive to UV-B for vitamin D induction), alongside use of a vitamin D insufficient diet (containing 200 IU/kg, approximately 10% the level of vitamin D as chow) [2, 5].

This study follows up on the previously published work of others highlighted above [5, 8, 11, 12], investigating the effect of UV exposure at levels comparable to terrestrial sunlight in a high fat diet (HFD) treated C57BL6 mouse model, with sham exposed mice receiving comparable HFD or low-fat diet (LFD) therein serving as controls Here we include female mice for assessment of weight gain and liver steatosis as a point of focus. This is necessary for a complete assessment of metabolic disease considering previous work excludes female mice [5, 8, 11, 12]. In turn this serves to untangle sex dimorphism potentiated through vitamin D and non-vitamin D mediated UV-driven mechanisms in supressing disease. As UV poses carcinogenic effect through the ability of shortest wavelengths to interact directly with DNA [4], human skin explants have also been incorporated into study to determine mechanistic effect locally in the skin in terms of double stranded DNA breaks and CPD’s with comparable doses of UV as used within in-vivo work. This permits a targeted analysis of the direct risks to skin against systemic benefits omitted in other studies [5, 8, 11–13].

## Materials and methods

### Mice

All experiments were performed according to the UK Home Office regulations under project licence PP4753477. Non-transgenic male and female C57BL6 mice were purchased from Charles River laboratories at 4 weeks of age. Mice were selected for each group in a randomised manner following purchase from supplier prior to application of diet and light regimen, no runts were included in the study. Upon entering the study mice were housed with normal 12 h light and dark cycles in type II polycarbonate cages with wire bar lids. Temperature between 19–21 °C and humidity between 40–60% within animal rooms was controlled. Mice were harvested at 20 weeks of age with blood and organs harvested within comparable timeframe between cohorts of UV exposed and unexposed male and female mice on high fat diet and low fat diet. This schedule was observed to avoid potential variations in cytokine and chemokine levels associated with circadian rhythm. Sample size was selected based on initial literature review which suggested between five to eighteen mice were needed per group to produce detectable changes. Mice were excluded from the study for failure to put on weight, or for other signs of poor health (weight loss or abrasions from shaving etc). Criteria was established before starting study and set out in the license PP4753477.

### Diet

**Fig. 1:**
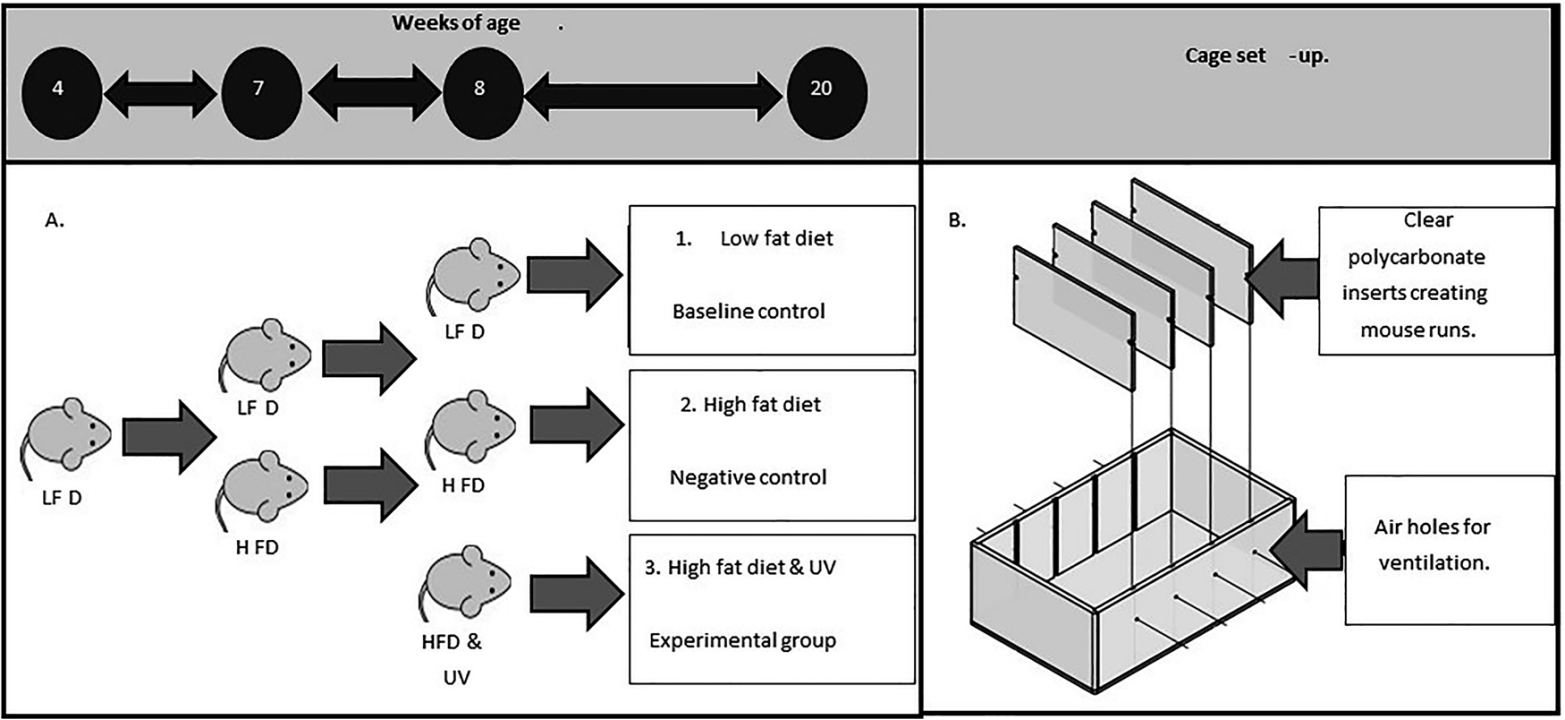
In-vivo study set-up. **A** Layout of in-vivo study over 20 week period incorporating male and female C57BL6 mice. Mice purchased at 4 weeks were placed on LFD then shaved at 7 weeks and placed on appropriate HFD (*n* = 20) or LFD (*n* = 10). Half of the mice on HFD (*n* = 10) had UV exposures starting at 8 weeks as described. Study groups were split evenly between male and female mice. **B** Cages used for exposure of male and female C57BL6 mice in-vivo. Modified cages built in house by engineers prevented mice from laying on top of each other and huddling together, while at the same time allowing mice to view each other meaning stress was reduced. Air holes within cages allowed for air flow and heat dissipation.

Diets were fed ‘ad-libitum’ with custom made low-fat diet (LFD) containing 5% fat from soya oil and a macronutrient comparable high fat diet (HFD) containing 23.4% fat from 20.7% lard, and 2.9% soya oil. Diet was obtained from Specialist Diet Services (SDS, Essex, UK) and supplied without added vitamin D but prepared to the same formulation as documented in previous work [5, 6, 8, 12], see Supplementary Table S1 (supplementary information). Each diet contained added calcium, magnesium and phosphorus to ensure normal calcium homeostasis [5]. In total, 60 (30 male and 30 female) mice were included over two consecutive intakes, with 30 (15 male and 15 female) in each intake. All mice were fed LFD for 3 weeks, with HFD being introduced to 67% of each intake, 10 male and 10 female mice thereafter (Fig. 1A). Failure of the 2^nd^ intake of male mice to gain weight meant that this cohort had to be excluded from study. The reasons for this are unknown, this left 45 mice (15 male and 30 female) in the final analysis.

### Ultraviolet exposure of mice

**Fig. 2:**
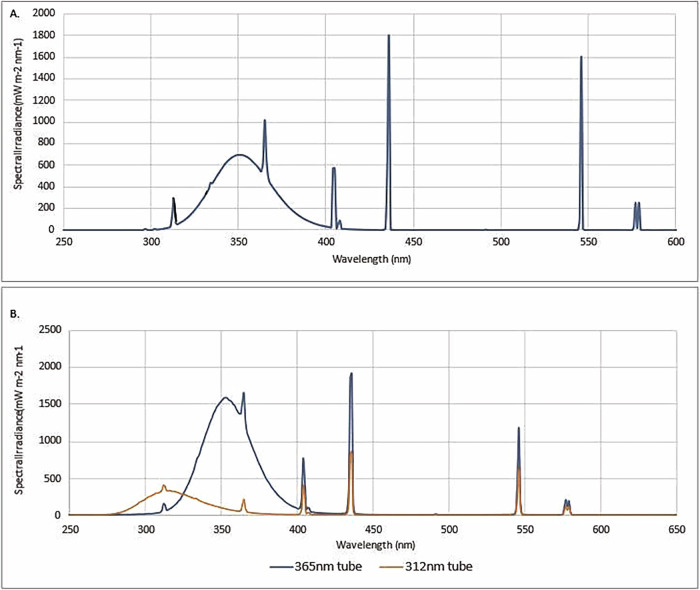
Spectral output of exposure lamps used. **A** Spectral irradiance of the CLEO light-source, with peak output at 350 nm, with 2% UV-B and 98% UV-A within the UV region. **B** Spectral irradiance of the Biosun, with peak output at 365 nm and 312 nm.

UV exposures were conducted using a facial tanning lamp (Summer Glo, Hapro HB-175, referred to hence forth as ‘CLEO’ light-source), incorporating four Phillips Cleo™ 15 W tubes with peak emission at 350 nm, with 2% UV-B and 98% UV-A over the UVR spectrum (280–400 nm), with 17% of the total output of the lamp in the visible region (Fig. 2A). Spectral irradiance was measured at the exposure distance in an environmentally controlled laboratory by double-grating spectroradiometer IDR300 (Bentham Instruments, UK) calibrated for spectral irradiance to the Physikalisch-Technische Bundensanstalt (PTB, Germany) traceable reference standards (Fig. 2A).

To ensure reproducible UV exposure to the mouse skin, at 7 weeks of age all mice were placed under general anaesthesia and an 8 cm^2^ area shaved between the base of neck and tail, with shaving being repeated every 7–10 days to prevent regrowth of fur. Exposures were conducted from 8 weeks of age, twice weekly for a period of 10 min totalling 24 exposures. Exposures were conducted by placing mice in type II polycarbonate cages with wire bar lids, modified with polycarbonate walls to create individual ‘lanes’ (Fig. 1B). This allowed group-housed mice to visualise/smell each other during exposures therein reducing stress; polycarbonate dividers also prevented mice from huddling during exposures [14, 15]. Mice were exposed 30 cm under the light-source delivering a dose of 1.83 J/cm^2^ UV-A and 0.04 J/ cm^2^ UV-B for each10-minute exposure. Groups of 5 female and male mice on LFD and HFD were sham exposed, in an identical exposure cage forming control groups. The remaining 5 female and male mice on HFD were treated with UV forming study group HFD/UV. Mice were exposed in groups, with males first, followed by cage washing then females to ensure pheromones within cage were kept at normal levels. Weights were obtained weekly 24 h after the second exposure, and mice were harvested at 20 weeks of age. Mice were visually assessed throughout the study for visible signs of oedema or erythema.

UV exposures were carried out at the same time in the day for all mice. Multiple exposure cage set-ups with identical light sources were used to avoid artifacts that may be experienced between groups due to differences in time of day or circadian entrainment.

### Oil Red O staining of liver samples

Liver samples were snap frozen in OCT via immersion in cold 2-Methylbutane maintained on dry ice. 10 µm sections were then cut onto glass slides and stored at 80 °C until use. To detect the presence of lipid bodies in liver sections the Oil Red O kit (ABCAM 150678) was used, and a modified protocol applied. Briefly, sections were first fixed in 10% formalin over 5 min, washed with deionized water then placed in propylene glycol for a further 5 min. Sections were then placed in Oil Red O solution for 10 min at 60 °C permitting lipid bodies within sections to stain red. Propylene glycol addition after staining permitted excess Oil Red O solution to be removed, washes with propylene glycol followed by water were carried out for a 3-min period.

### Masson’s trichrome staining

The Masson’s trichrome stain kit (ABCAM ab150686) was used as per manufactures protocol. Briefly, liver samples were collected, frozen in OCT and 10 µm sections were collected via cryostat and placed on glass slides. To detect the presence of collogen deposits sections were first treated with Bouin’s Fluid at 56–64 °C for 60 min followed by a 10-min cooling period and subsequent washing under tap water. Sections were then stained in working Wiegert’s Iron Haematoxylin for 5 min, prepared according to manufacturer’s instructions, then stained with Biebrich Scarlet/Acid Fuchsin Solution and Phosphomolybdic/Phosphotungstic Acid Solution for 10–15 min with a final wash in aniline Blue Solution for 5–10 min and subsequent rinsing in distilled water to remove excess stain in between. After staining, the process was completed via immersion of slides in 1% acetic acid for 3–5 min prior to drying of sections and mounting on slides.

### Cytokine arrays

Cytokine arrays (ABCAM, AB193660) were carried out in triplicate on HFD and HFD/UV liver specimens that had undergone lysis and protein quantification via BCA assay (Pierce 23225). 500 µg protein lysate was used on blocked membranes overnight followed by successive washes and staining with primary and secondary array antibodies as outlined in manufacturers protocol, as with Western blotting threshold of cytokines were detected in blots via chemiluminescence technique.

### Vitamin D ELISA assay

To detect upregulation in vitamin D due to exposure to CLEO lamp, a competitive 25(OH)D vitamin D ELISA assay was conducted (immunodiagnostic systems, AC-57SF1), shown in previous studies [5]. 300 µg of liver lysate was used to complete the assay, for blood serum test 1000 µg of serum was used. All mice in trial were processed and used to generate end ELISA data. For visualisation of ELISA capture antibody was added for 1 h preceding chemiluminescence detection.

### Cell culture and ex-vivo punch biopsies

Primary neonatal keratinocyte cell lines (FSK) and ex-vivo skin punches were obtained from donor foreskins from white skin neonates (1–3 weeks of age) received after routine circumcision. Prior to sample receipt and processing, informed consent was obtained from the child’s parent or legal guardian, and ethical approval put in place under the South-central Berkshire B ethics committee 22/SC/0411, IRAS ID 31832. As outlined previously [16], to ensure sterility prior to receipt tissue was placed in transport medium containing high glucose DMEM (SLS 16-405-CV) supplemented with 10% FBS (Gibco 10500064), penicillin (P4333, SIGMA), streptomycin and amphotericin (R01510, Thermo-Fisher). Upon receipt, tissue was processed as outlined previously for in vitro cell exposures or kept whole for ex vivo tissue exposures [16, 17]. Briefly, for the cell exposures tissue was cut into oblongs approximately 5–10 mm in size and digested overnight at 4 °C with 0.5 mg/ml liberase dispase (5401089001, ROCHE) in CnT-07 keratinocyte medium (CELLnTEC). Following digestion, the epidermis was peeled from the dermal layer, transferred to trypsin–EDTA, and mechanically dissociated to form a single-cell keratinocyte suspension (FSK). Following pelleting, FSK were resuspended in CnT-07 keratinocyte medium (CELLnTEC) and seeded into Petri dishes first coated with type one collagen and fibronectin derived from human plasma. FSK monolayers were grown until 95% confluent, media was then removed and cells rinsed with HBSS −/− (Appleton Woods, CSR151). Three millilitres of pre-warmed PBS +/+ (Corning, 10500064) were then added and cells exposed to UV as described below. Cell cultures and tissue were not tested for mycoplasma contamination.

### Ultraviolet exposure of in vitro and ex vivo samples

Cell culture and punch biopsies were irradiated with a single exposure under the same conditions as the mice in PBS+/+ with a positive control attained in the BIOSUN UV device (Vilber Lourmat™). The BIOSUN houses four 30 W UV-A tubes with peak emission at 365 nm, and two 30 W UV-B tubes with peak emission at 312 nm (Fig. 2B). The positive control was irradiated with 9 J/cm^2^ UV-A and 1 J/cm^2^ UV-B. The BIOSUN incorporates live dose and temperature monitoring ensuring consistent dose delivery. Negative controls were retained within the same environment with the absence of UV exposure by covering with black tape.

### Western blotting

Following UV exposure of the in vitro cells, plates were scraped, cells collected, and quantification of protein yields were attained via standard BCA technique (Pierce, 23225). For western blotting 10 µg of protein per sample lane was loaded into a 12% separating gel with a 4% stacking gel. The gel was run at 120 V for 1 h and transferred to PVDF membrane via a semi-dry transfer system (Trans Blot Turbo unit-Bio-Rad). After transfer the membrane was then blocked with 10% milk powder in Tris-Buffered Saline-TWEEN (TBST) for 1 h at room temperature. Primary antibody for DNA damage assessment phospho-H2AX (Cell Signalling, 9718S) was then incubated overnight at room temperature in TBST with 10% milk powder at a dilution of 1:5000. Blots were counter-stained with the control antibody GAPDH 1:5000 (SANTA-CRUZ, SC25778) to ensure correct loading. Primary antibodies were detected via chemiluminescence with donkey anti-rabbit antibody (SANTA CRUZ, SC2313) at a 1:10,000 concentration following incubation for 1 h at room temperature.

### Assessment of DNA damage within ex-vivo tissue samples

Cyclo-butane pyrimidine dimer (CPD) staining was performed using punch biopsies of the UV exposed ex vivo tissue. Samples were frozen in OCT via immersion in cold 2-Methylbutane maintained on dry ice. After preservation samples were cut into 10 µm sections placed on glass slides and stored at −80 °C until use. For staining primary antibody (2B scientific, CAC-NM-DND-001) were added to the sections at a 1:100 concentration followed by overnight incubation at 4 °C in a humidified chamber. This preceded preparation of the sections for antibody staining via preservation consisting of 10% formalin for 10 min, followed by blocking in TBS containing 0.3% Triton X-100, 5% donkey serum buffer for 1 h and denaturation of tissue in 2 molar hydrochloric acid for 30 min*.*

### Statistical methods

For statistical analysis weight gain was analysed via Tukey test, with mixed effects analysis permitting each average weekly weight taken from cohorts to be cross-compared against one another, this permitted detailed comparison of weight gain over study duration, not only at endpoint. Percentage weight gain was also calculated and displayed from average weight recorded. Oil red O staining and vitamin D status were assessed via ordinary one-way ANOVA. Cytokines were quantified from average florescence attained in 3 mice deriving t-Test with F-test to test variance of populations. The florescence was then represented in the text as a fold change. For in vitro analysis of DNA damage in keratinocyte monolayers one way ANOVA was used. For every figure generated statistical tests were justified as appropriate by departmental statisticians, with data meeting the assumptions of the tests. An estimate of variation in the group data was recorded with standard deviation used to represent variance between groups in figures generated where applicable.

For in vivo and in vitro work blinding was not employed. All data were analysed for statistical variation in GraphPad Prism™ software, except for cytokine arrays (Microsoft Excel™). Prior to evaluation on Prism and Excel, Image-J (FIJI) was used to cross compare area change florescence recordings. For all analyses, significance was set at *p* < 0.05. Histological staining, cytokine array and vitamin D analysis used prior to statistical analysis downstream techniques were carried out following harvest of last mouse from designated cohort, with all harvested mice from each gender processed in unison.

## Results

### Low dose UV exposure curbs weight gain in C57BL6 mice on HFD at a comparable rate in male and female mice by study endpoint

**Fig. 3:**
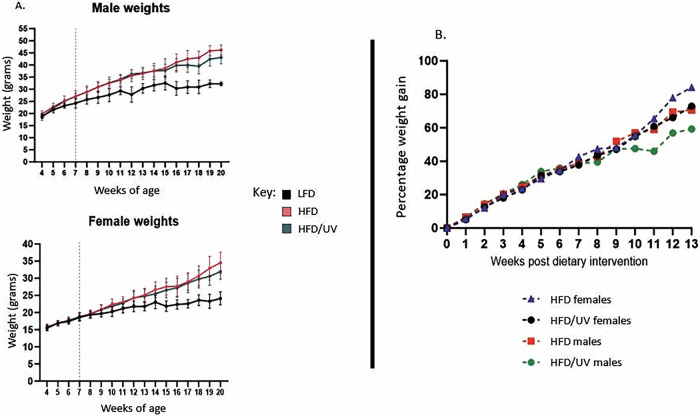
Weight retention in mice over study period. **A** Weights attained from male and female mice on LFD, HFD and HFD/UV from 4 weeks of age (buy-in date of mice). HFD gave significant effect from week 6 in males (*p* = 0.0159). In females this effect was present from week 13 (*p* = 0.0134 for HFD, *p* = 0.0319 for HFD/UV). No significant differences between weights of mice on HFD or HFD/UV were visualised throughout the course of the work (*p* > 0.05), however at study endpoint a non-significant difference of 6.6% males and 7.3% females were recorded between HFD and HFD/UV treated mice. **B** Data expressed as percentage weight gain.

HFD induced significant weight gain in mice compared to the LFD controls with end study values of less than *p* = 0.0001 recorded for both genders regardless of sex or UV exposure. For male mice this initiated at week 6 in HFD/UV treated males (*p* = 0.0159) and 9 in HFD males (*p* = 0.0185). In females, regardless of UV exposure, weight gain became significant at week 13 (HFD/UV *p* = 0.0319, HFD *p* = 0.0134) (Fig. 3A).

Comparison of HFD and HFD/UV provided evidence of a non-significant difference in weight gain when UV was administered (*p* = 0.830 in males and *p* = 0.4691 in females at study endpoint). In males this was present within weeks 16–20 and females within weeks 18–20. Data are also presented as percentage weight gain over time (Fig. 3B).

### Lipid droplet build-up in the livers of mice treated with HFD is reduced with UV exposure

**Fig. 4:**
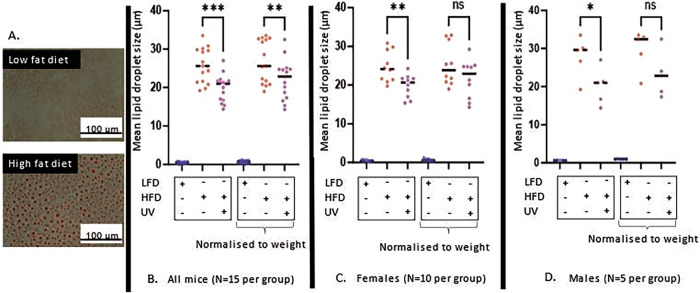
Liver lipid retention in mice as denoted by oil red 'O' staining. **A** Visual representation of lipid retention in mice subjected to LFD and HFD with lipid droplets in red stained with Oil red ‘O’. **B** Data derived from ImageJ for lipid droplet size in male and female mice combined. Data for all mice showed a significant difference in lipid droplet size (*p* = 0.0002) before normalisation to end weight, and *p* = 0.0033 after normalisation to end weight. **C** Data derived from ImageJ for lipid droplet size for female mice only. Difference in lipid droplet size was significant in female mice (*p* = 0.0073) before normalisation to weight and borderline significant (*p* = 0.0570) after normalisation to weight. **D** Data derived from ImageJ for lipid droplet size for male mice only. Difference in lipid droplet size was significant in male mice (*p* = 0.0207) before normalisation to weight and borderline significant (*p* = 0.0524) after normalisation to weight.

HFD mice had induced lipid retention in the liver at study endpoint regardless of sex (Fig. 4A). When all mice were considered, a significant drop in lipid droplet size was observed in HFD/UV mice when compared to HFD only mice (*p* = 0.0002): a reduction in lipid droplet size by 5 μm (30%) was observed at study endpoint after UV exposure. As UV exposed mice showed a trend towards lower weight gain, weight was used to normalise data. Here significant difference was still reported (*p* = 0.0033), with a reduction in lipid droplet size 4 μm (18%) at study endpoint in UV exposed groups (Fig. 4B).

Considering each sex, the difference in lipid droplet size after UV exposure was significant in males (*p* = 0.0207) and females (*p* = 0.0073), with HFD males retaining 37% more lipid in the liver compared to females (Fig. 4C, D). However, after normalisation to weight only borderline significance was seen for females (*p* = 0.0570) and for males (*p* = 0.0524). For females, a reduction in lipid droplet size from 5 μm (25%) was recorded, with 4 μm after normalisation to weight (14%) (Fig. 4C). For males, a reduction in lipid droplet size from 8 μm (35%) was recorded, with 7 μm recorded after normalisation to weight (30%) (Fig. 4D).

### At the end of the study, after week 20 mice display MASLD/simple steatosis with no presence of fibrosis indicating MASH

**Fig. 5:**
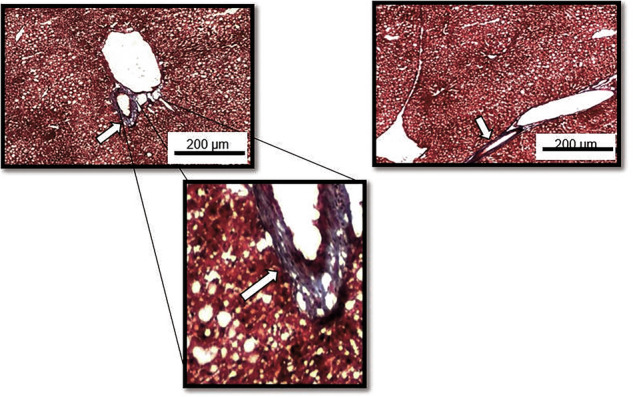
Masson’s Trichrome stain evaluating the presence of collogen build-up (blue) indicating scarring. No collogen was visualised in HFD (left) and HFD/UV (right) treated mice except around arteries where commonly found (white arrows).

Masson’s trichrome staining suggests the mouse model is representative of simple steatosis (MASLD), with collogen identifiable only within artery walls, and not main liver architecture (Fig. 5) as is present with MASH (metabolic dysfunction-associated steatohepatitis).

### No difference in inflammatory response is recorded between HFD and HFD/UV mouse cohorts in cytokines surveyed

Results suggested no indication that significant inflammation was present in the mouse cohorts, via cytokine analysis (*n* = 3 per cytokine for each sex). Females on HFD display 91% of cytokines at non-significant levels when compared to HFD/UV exposed females, and males show 98% of cytokines at non-significant level when compared against UV exposed cohorts. Of the cytokines surveyed, 4-1BB and IL-11 decline in females surveyed when UV exposure is administered, and FC-gamma RIIB, MMP2 and Trance are upregulated. In males, only CD36 is recorded as downregulated, see Supplementary Table S2 (Supplementary Information).

### Comparable levels of 25(OH)D were recorded in liver lysate between cohorts. However, circulatory vitamin D varied in response to sex, diet and UV exposure

**Fig. 6:**
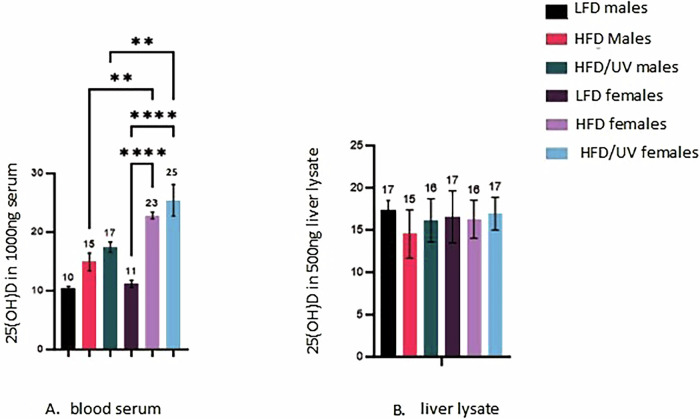
25(OH)D status of mice at end of study. **A** A rise in 25(OH)D was recorded in males and females through HFD regardless of UV treatment when compared against those on LFD. In males this was non-significant (*p* ≥ 0.999), and in females this was significant (*p* ≤ 0.0001). When genders were compared 25(OH)D was significantly elevated in females in HFD (*p* = 0.0076) and HFD/UV (*p* = 0.0081) exposure groups when compared against male cohorts. HFD/UV showed a non-significant trend (*p* = 0.5590 males and *p* = 0.3606 females) towards higher level of vitamin D than HFD. **B** Liver samples assayed showed comparable level of 25(OH)D regardless of diet or exposure type.

For assessment, every mouse in the study had blood and liver samples surveyed. Blood plasma showed significant differences in 25(OH)D level pertaining to sex, with HFD females showing significant elevation in 25(OH)D compared to HFD males (*p* = 0.0076) (Fig. 6A). UV exposure also mediated a non-significant increase in 25(OH)D in males (*p* = 0.5590) and females (*p* = 0.3606). All mice on LFD displayed lower vitamin D status than mice on HFD. This was non-significant in males (*p* ≥ 0.999) and significant in females (*p* ≤ 0.0001). Liver lysate derived a comparable level of vitamin D across all treatment groups (Fig. 6B).

### Use of CLEO light source on primary cell monolayers and ex-vivo tissue induced comparable levels of CPD dimers and non-significant upregulation in DNA damage as compared to unexposed controls

**Fig. 7:**
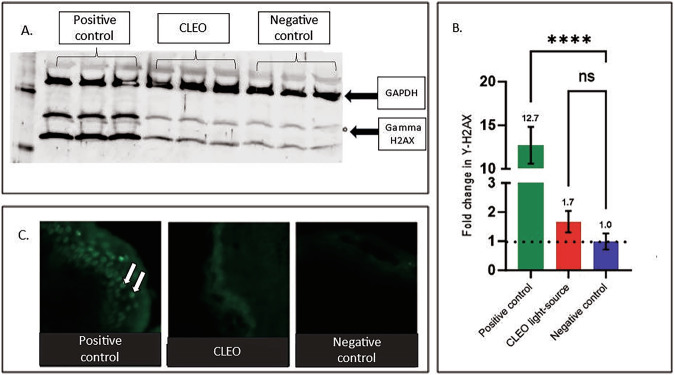
DNA damage assessment of exposure lamp in human skin explant and keratinocyte monolayers. **A** DNA damage assessment of primary skin cells. Here Western blot was used for assessing γ-H2AX induction, representing double stranded breaks after UV light exposure in neonatal keratinocyte monolayers (3 different donors) with GAPDH as a loading control. Loaded as positive control first (Biosun (1J/cm2 UV-B 9J UV-A), Cleo light-source (forming experimental group) (UV-A 20 J/cm^2^ and UV-B 0.4 J/cm^2^) and negative (unexposed) control. **B** Assessment of western blotting bands via ImageJ analysis. Here results are represented as fold change with positive, CLEO test and negative respectively. **C** Cyclobutane pyrimidine dimers (CPDs), denoting charesteristic signiture mutantions induced by UV exposure. CPD staining in explants of neonatal skin sections with positive control (Biosun), Cleo and negative control, white arrows point to examples of CPD staining.

Western blotting revealed a non-significant elevation in double stranded breaks (1.7-fold change) from exposure to CLEO lamp when compared with unexposed controls in keratinocyte monolayers (*p* = 0.7789) (Fig. 7A, B). By comparison, the positive control induced 12.7-fold more double stranded breaks than unexposed controls (*p* = <0.0001). Assessment of CPDs suggested that exposure to the CLEO lamp induced no more CPDs than unexposed controls, with no CPDs detectable (Fig. 7C).

In addition, mice displayed no traits akin to adverse reactions from UV exposure such as oedema of the skin and reddening of the skin.

## Discussion

Non-significant trends visualised in this project support previous evidence and suggest that UV exposures akin to sunlight can slow weight gain [5, 6], supporting a need for larger studies to further validate these findings. When percentage weight gain is considered (Fig. 4C), the 10% difference in females and 12% difference in males due to UV exposure recorded at study endpoint mirrors data by Geldenhuys et al. [5]. In their work, a 10% reduction in weight gain was seen in male mice when UV-B rich FS40 lamp exposure was used, incorporating 0.04 J/cm^2^ UV-A and 0.1 J/cm^2^ UV-B [5, 11, 12]. This suggests the weight suppressing effect from UV exposure is comparable across sexes.

Although our work supports this effect in young healthy mice at the point of HFD/UV exposure it contradicts others who assess the effect of UV exposure on metabolic insult outside these conditions [5–7, 11, 12]. This is evident considering, the work of Fleury et al. and Teng et al. in already overweight mice and aged mice respectively, with both studies reporting no significant effect on weight gain [11, 12]. The data from Fleury et al. is particularly interesting through use of the same CLEO light source, delivering similar exposures of UV-B 1 kJ/m^2^ & UV-A 20 kJ/m^2^ twice weekly. Our study showed that UV-mediated reduction of weight gain is not derived until at least 9 weeks after exposures are started (Fig. 3). By comparison, it is hypothesized that a 6-week regimen timeframe by Fleury et al. may not be long enough to witness positive effect from UV exposure. A review of the study by Teng et al. also suggests the use of single housing may alter the response of mice through stress [12, 18].

In our study female mice were significantly more responsive to 25(OH)D from diet (Fig. 6). This could contribute toward lower lipid droplet size in HFD treated female mice when compared against HFD males (Fig. 4). The HFD/UV exposures in this study also non-significantly alter 25(OH)D by 2 ng/μl when compared against HFD groups. Considering these non-significant trends and the fact that liver steatosis was slowed in males to a comparable level as females before and after normalisation to weight in UV exposed mice, alongside significant difference in vitamin D status through diet this supports previous evidence that other mediators than vitamin D may aid in potentiating this effect [5, 11, 13].

Bias toward young male mice means data needs to be followed up in both female and aged mice [5, 11, 12]. This is imperative as liver disease is reported to be worse in females after menopause, owing to drops in oestrogen affecting uptake of vitamin D [19, 20]. Approaches to strengthen experimental models could be adopted through dietary intervention or ovariectomy to induce appropriate hormonal changes as female mice undergo age-related decline in fertility differently to humans [9, 21, 22].

Further studies could consider the irreversible effects on the liver that occur as part of later metabolic disease such as MASH and fibrosis [23]. In our work, cytokine array and Masson’s trichrome staining evidence indicated that only simple steatosis is present (Fig. 5 & Supplementary Table S2, Supplementary Information). A balance between an appropriate in vivo model that mimics disease progression, and timeframe needed to reach study endpoint reliably is required. This is highlighted here as the removal of methionine from diet, necessary to induce collogen build-up in an appropriate timeframe, induces steatosis through non comparable mechanisms with respect to western diet [24]. Additionally, this affects mitochondrial uncoupling, and responses to hunger related adipokines such as leptin understood to be implicit in weight related effects in HFD models [25].

In consideration of non-vitamin D mediated mechanisms that may also alter liver disease and weight gain, the potent vasodilator nitric oxide has also been cited [5, 8, 11]. This is demonstrated through the effect of topical application of the potent nitric oxide down regulator CPTIO on the skin of UV exposed mice, preventing positive effects on the liver from UV exposure, and application of the potent nitric oxide liberator SNAP on the skin of sham treated mice which garner beneficial effects on the liver [5, 8, 11]. Work outside of UV-based studies also suggests this association with increased dietary nitrite intake [26] and nitric oxide hydrogels subcutaneously inserted below mouse skin mediating positive effect, adding further credence to interplay between the liver and nitric oxide [27].

Considering study limitations, light exposure regimen and endpoints longer than the 20-week timeframe may also be addressed in further work in strains of mice other than C57BL6. Firstly, incorporating lamp types that exclusively emit UV-A to mice or visible light also cited to induce lower threshold of effect [28]. This is important given all studies to our knowledge in this area have lamps with at least trace UV-B emissions and in some studies – with very substantial UV-B emission, as well as emission of visible light. This would be useful to gain a fuller mechanistic insight into these effects. Additionally, it would be useful to investigate if repeated exposures with lower recovery time of skin magnify positive results or carry limited effect. In carrying out this work local damaging effect from UV on the skin, particularly from UV-B that carries direct effect in terms of DNA damage should also be documented, as presented within this work, with data suggesting that exposures from the CLEO lamp would provide minima adverse effect to the skin in terms of direct DNA damage (Fig. 7).

### To conclude

Evidence is presented that UV exposure akin to terrestrial sunlight derives positive impact on the liver and adipose tissue in young male mice, in agreement with previously published evidence. The study demonstrates this response in female mice highlighting the differences in vitamin D status between sexes. However, weight amelioration through UV exposure does not show sex dimorphism at study end point, further supporting the hypothesis that mediators other than vitamin D are involved.

Further research may broaden understanding of mechanisms by focusing on the hypothalamus-pituitary axis (HPA) which is implicated in the development of metabolic disease and shown to be altered by the action of UV light on the skin and eyes in C57BL6 mice [29–31]. Although HPA modulation from skin derived neurotransmitters exhibits greatest effect from UV-B and UV-C [30], [31], recently it has also been demonstrated that UV-A exposure induces lower threshold response through direct action and from reactive intermediates such as NO. In considering the action of UV-B, the by-products lumisterol and tachysterol uniquely generated in the skin after exposure are also worthy of assessment due to recent evidence of the protective capability against DNA damage and oxidative stress, with antifibrogenic and anticancer activity [32].

Further work is necessary to build on downstream mechanisms. Here work should give particular focus to aged and/or already overweight mice, with inclusion of female cohorts, and later stage liver disease by use of a western diet or a fructose/cholesterol rich high fat diet, currently underrepresented in the published literature. In addition, isolation and use of longer UV wavelengths and/or visible light, proffered to also induce nitric oxide [28], will further elucidate the most effective preventative or therapeutic interventions.

## Supplementary information

S1

S2

## Data Availability

The datasets generated during and/or analysed during the current study are available from the corresponding author on reasonable request.
