# Supplementary material for: Ultraviolet exposure of mice fed a high fat diet reduces weight gain and markers of liver disease progression
Source: Int J Obes (Lond). 2025 Apr 28;49(7):1373–81. doi: 10.1038/s41366-025-01779-5 (PMC12283362; doi:10.1038/s41366-025-01779-5)
Supplement: Supplementary file 1 — S1 [file 41366_2025_1779_MOESM1_ESM.pdf]

Table S1

Number	Cytokine	Fold change (females)	P value (females	F Test (females)	Significant	Fold change (males)	P-value (males)	F-test (males)	Significant
1	**4-1BB**	0.5	0.007	0.76	Yes	1	0.959	0.77	
2	**AXL**	1	0.904	0.3		1.1	0.877	0.38	
3	**CD30 L**	1	0.816	0.32		0.8	0.131	0.62	
4	**CD36**	1	0.989	0.16		0.5	0.021	0.22	Yes
5	**CD40 ligand**	1.3	0.161	0.15		0.7	0.06	0.72	
6	**Chordin**	1.1	0.777	0.27		0.7	0.296	0.03	
7	**CRG-2**	1	0.714	0.49		1.1	0.73	0.16	
8	**CTACK**	0.9	0.299	0.14		1.4	0.597	0.2	
9	**CXCCL1**	1	0.795	0.56		0.7	0.275	0.1	
10	**EOTAXIN -1**	0.8	0.393	0.74		1.4	0.526	0.27	
11	**EOTAXIN-2**	1.1	0.591	0.66		1.5	0.664	0.02	
12	**FC GAMMA RIB**	1.7	0.006	0.71	Yes	0.6	0.181	0.84	
13	**FN14**	1.2	0.591	0.75		0.7	0.693	0.31	
14	**Galectin-1**	1.1	0.712	0.5		0.7	0.263	0.22	
15	**GAS1**	1.1	0.531	0.24		0.8	0.253	0.83	
16	**GAS6**	1	0.798	0.97		0.8	0.19	0.74	
17	**GM-CSF - blot 2**	1.1	0.357	0.28		1.1	0.406	0.35	
18	**Granzyme B**	1.3	0.2	0.31		0.6	0.287	0.3	
19	**GSCF - blot 2**	0.9	0.725	0.43		1	0.852	0.76	
20	**ICAM-1**	0.8	0.146	0.25		0.9	0.354	0.78	
21	**IFN-GAMMA**	1.1	0.316	0.49		1	0.987	0.27	
22	**IGF-1**	1.2	0.313	0.65		0.5	0.053	0.19	
23	**IGFBP2**	1.2	0.521	0.46		0.6	0.067	0.07	
24	**IL-1 alpha**	1	0.524	0.85		0.8	0.301	0.28	
25	**IL-1 beta**	1	0.977	0.02		0.8	0.515	0.2	
26	**IL-10**	1	0.997	0.38		1.2	0.609	0.06	
27	**IL-11**	0.6	0.025	0.93	Yes	1.1	0.784	0.03	
28	**IL-17B**	0.7	0.026	0.27	Yes	1	0.905	0.19	
29	**IL-17E**	1	0.119	0.8		1.3	0.237	0.47	
30	**IL-17F**	1	0.094	0.36		1.1	0.734	0.44	
31	**IL-21**	1.2	0.54	0.46		0.9	0.435	0.36	
32	**IL-28**	1.1	0.801	0.11		0.9	0.399	0.25	
33	**il-2r alPHA**	0.8	0.309	0.97		0.8	0.354	0.23	
34	**IL-6**	1	0.762	0.69		1.4	0.285	0.76	
35	**IL-6R**	1	0.857	0.79		0.8	0.121	0.37	
38	**IL-9**	1	0.708	0.71		1.4	0.113	0.2	
39	**ITAC**	1.3	0.054	0.51		0.7	0.194	0.67	
40	**JAM-A**	1.5	0.12	0.03		0.8	0.095	0.85	
41	**KC - blot 2**	0.9	0.203	0.67		1.1	0.62	0.25	
42	**MCP-1**	1	0.939	0.09		0.9	0.667	0.84	

43	**MCP5 - blot 2**	1.2	0.417	0.88		1	0.966	0.6	
44	**MMP-2**	1.6	0.005	0.91	Yes	0.5	0.129	0.42	
45	**OPN**	1.3	0.201	0.88		0.8	0.629	0.9	
46	**PRL**	0.9	0.51	0.07		1	0.772	0.2	
47	**P-SELECTIN**	1.1	0.508	0.51		1.1	0.732	0.03	
48	**PTX3**	0.9	0.351	0.45		1.2	0.485	0.2	
49	**RANTES**	1.1	0.552	0.9		1.3	0.699	0.03	
50	**TIMP-2**	1.3	0.066	0.21		0.6	0.372	0.8	
51	**TNF-ALPHA**	1.1	0.674	0.38		1.1	0.761	0.45	
52	**TRANCE**	1.2	0.039	0.78	Yes	0.5	0.392	1	
53	**TREM 1**	1.3	0.261	0.12		1.1	0.347	0.41	
54	**TROY**	1.4	0.023	0.44		0	0.974	0.93	
55	**TWEAK**	1.5	0.195	0.05		0.9	0.986	0.75	
56	**VCAM1**	1.2	0.381	0.59		1.2	0.634	0.23	
57	**VEGF R1**	1.2	0.349	0.66		0.8	0.312	0.4	

Table S1. Cytokine array results for male and female mice showed simple steatosis was present after administration of high fat diet, with over 90% of cytokines showing non-significant deviation from sham exposed controls on high fat diet. CD36 was significantly upregulated for males. For females significant differences were seen for 4-1BB, FC-Gamma RIIB, IL-11, ITAC and trance. Cytokines displayed were selected due to a minimum cut-off of 200 in recorded florescence. Florescence values were averaged and displayed as fold change with F-test and P-values recorded from original florescence recordings.
