# Supplementary material for: Ultraviolet exposure of mice fed a high fat diet reduces weight gain and markers of liver disease progression
Source: Int J Obes (Lond). 2025 Apr 28;49(7):1373–81. doi: 10.1038/s41366-025-01779-5 (PMC12283362; doi:10.1038/s41366-025-01779-5)
Supplement: Supplementary file 2 — S2 [file 41366_2025_1779_MOESM2_ESM.pdf]

Table S2.

**Ingredient**	**Inclusion (%) Low fat diet**	**Inclusion (%) High fat diet**
Calcium carbonate	2.52	2.52
Casein	20	20
Cellulose	5	5
Choline chloride (50%)	0.38	0.38
Dicalcium Phosphate	5.1	5.1
Lard	0	20.7
Magnesium oxide	0.17	0.17
Maize starch	35.87	17.27
Maltodextrin	13.2	13.2
Methionine	0.3	0.3
Mineral mix	0.14	0.14
Potassium citrate	0.15	0.15
Potassium dihydrogen phosphate	0.76	0.76
Potassium sulphate	0.16	0.16
Salt	0.26	0.26
Soya oil	5	2.9
Sucrose	10	10
Vitamin mix - no added vitamin D	1	1

Nutrition information

Additional information:

Comparable to that used in work undertaken by Shelly Gorman et al, with the following exceptions:

- Maize starch used in place of wheat starch.
- Soya oil used in place of canola oil.
- 50% Choline chloride used in place of 75% choline chloride – starch used to balance this change.
